# Anomalous elastic properties across the γ to α volume collapse in cerium

**DOI:** 10.1038/s41467-017-01411-9

**Published:** 2017-10-31

**Authors:** Magnus J. Lipp, Zs. Jenei, H. Cynn, Y. Kono, C. Park, C. Kenney-Benson, W. J. Evans

**Affiliations:** 1 Lawrence Livermore National Laboratory, Livermore, CA 94550 USA; 2 HPCAT, Geophysical Laboratory, Carnegie Institute of Washington, Argonne, IL 60439 USA

## Abstract

The behavior of the *f*-electrons in the lanthanides and actinides governs important macroscopic properties but their pressure and temperature dependence is not fully explored. Cerium with nominally just one 4*f* electron offers a case study with its iso-structural volume collapse from the γ-phase to the α-phase ending in a critical point (*p*_C_, *V*_C_, *T*_C_), unique among the elements, whose mechanism remains controversial. Here, we present longitudinal (*c*_L_) and transverse sound speeds (*c*_T_) versus pressure from higher than room temperature to *T*_C_ for the first time. While *c*_L_ experiences a non-linear dip at the volume collapse, *c*_T_ shows a step-like change. This produces very peculiar macroscopic properties: the minimum in the bulk modulus becomes more pronounced, the step-like increase of the shear modulus diminishes and the Poisson’s ratio becomes negative—meaning that cerium becomes auxetic. At the critical point itself cerium lacks any compressive strength but offers resistance to shear.

## Abstract (web summary)

The origin of the volume collapse of cerium, the only elemental metal with a critical point in the solid phase, remains elusive. Here the authors show that, near the critical point, the f-electrons make cerium lose its compressive strength while maintaining a finite shear strength—which makes cerium unexpectedly auxetic.

## Introduction

In the rearranged periodic table of Smith and Kmetko^1^, cerium takes the upper left corner sitting on the diagonal which delineates the boundary between itinerancy and localization, bonding and magnetic moment formation of the *f*-electrons. Elements sitting on this diagonal are highly susceptible to small perturbations resulting in a high number of crystallographic phases (four for cerium at atmospheric pressure), are highly reactive, or exhibit instabilities^2^. Other elements on this diagonal are iron and plutonium (Pu), the latter with even more phases than cerium. The behavior of the one *f*-electron in Ce is responsible for the volume dependence under pressure which contracts abruptly by ~15% at ambient temperature (RT) when the pressure reaches ~0.75 GPa^3^. This volume collapse (VC) announces itself by a gradual softening of the bulk modulus in the γ-phase which reaches a minimum at the moment of the transition and then rises again in the α-phase with a discontinuous change in slope^3–5^. The crystallographic structure remains, unparalleled among the elements, but other than that the two fcc phases are quite different. While the γ-phase shows the magnetic susceptibility *χ* of a trivalent atom, the higher pressure α-phase starts out with just one fifth of the γ-phase *χ*-value and then loses even more^6, 7^ hinting at tetravalency. This scenario also plays out at higher temperatures but now the transition requires more pressure and the volume change Δ*V* is reduced. It finally ends in a critical point at the critical pressure *P*_C_ ~ 1.5 GPa and the critical temperature *T*_C_ ~ 480 K^8–11^ with Δ*V* = 0 where the isothermal bulk modulus vanishes (*B*_T_ = −*V*(*∂p/∂V*)_T_ = 0). Remnants of this behavior can be observed even beyond the critical point since *B*_T_ still exhibits a non-vanishing minimum that separates the two types of solid. Now, however, the pressure derivative *(∂B*_T_/*∂P*) is continuous compared to the abrupt change below the critical point. With increasing temperature, the minimum becomes less pronounced, wider, and shallower^10^. Nevertheless, it appears to continue into the melt separating a γ-type (low density) from an α-type (high density) liquid^12, 13^.

This peculiar behavior has attracted a large theoretical effort—almost from day one after its discovery by Bridgman^3^ in 1927. Linus Pauling suggested that the VC and concomitant decrease of the magnetic susceptibility was caused by the promotion of the *f*-electron to a bond-forming orbital^14^. The spin–orbit coupling of the *f*-electrons in the lanthanides via Hund’s rules had explained the paramagnetism of lanthanide ions in salts very well^15^, one of the early triumphs of quantum mechanics. It was only natural to assume that the destruction of the magnetic properties was caused by the promotion of the *f*-electron. Beyond the paramagnetism that is directly derived from the *f*-electrons in the trivalent ions, the compression behavior is also strongly affected by them but the systematics are more complex. Praseodymium—with two *f*-electrons—also suffers a volume collapse, albeit at a much higher pressure of 20 GPa^16^, whereas neodymium’s (3 *f*-electrons) equation of state (EOS) evolves without one^17^. Over the years, the promotional model of Zachariasen–Pauling gave way to many others (see ref. ^18^ and references therein).

The current two front-runners explaining the VC mechanism are the Hubbard–Mott and the Kondo VC models^19–21^. The possible disappearance of the total angular momentum *J* points to the conjecture that the localized 4*f* electron becomes itinerant, undergoing a Mott transition^19^. In the other scenario, the 4*f* electron remains localized and the VC is caused by the onset of screening of the *f*-electron magnetic moment by the conduction band electrons, the Kondo mechanism^20, 21^. Both models attempt to quantify the VC by calculating the free energy and from there the EOS or phase diagram^22, 23^. Indeed, both models can interpret the EOS above the critical point^10, 24^ and the controversy continues.

Since the elastic moduli *C*_*ij*_ are the second derivatives of the free energy with respect to strain and the isothermal bulk modulus *B*_T_ the volume derivative of the free energy, they offer a connection to electronic structure calculations. *B*_T_ can be found experimentally by X-ray diffraction of polycrystalline samples. The *C*_*ij*_ can be studied with ultrasonic waves impinging on oriented single crystals. Measuring the transverse and longitudinal ultrasonic waves *c*_T_ and *c*_L_ in polycrystalline samples, one can obtain the pressure dependence of the adiabatic bulk modulus *B*_S_ and the shear modulus *G* ^5, 25–28^. Previous studies at RT also derived the Debye temperatures *θ*_γ_ and *θ*_α_ in each phase^5, 27, 28^. A significant part of the total entropy change Δ*S* across the volume collapse given by the Clausius–Clapeyron relation *∆S* = Δ*V·*d*P/*d*T* apparently originates with the lattice contribution Δ*S*_vib_ = 3*k*_B_·ln(*θ*_γ_/*θ*_α_)^4, 5,25–28^. The actual size is still under debate theoretically^29–31^ and experimentally^4, 5, 26–28, 32^. *∆S* is almost equal to *∆S*_J_ = *k*_B_ ln[(2*J*_γ_ + 1)/(2*J*_α_ + 1)] with *J*_γ_ = 5/2 and possibly *J*_α_ = 0 as the Hund’s rule moments in their respective phases. The assumption *J*_α_ = 0 is very appealing since it explains the 80% drop of the magnetic susceptibility across the VC^6, 7^ but it does not leave room for a sizable lattice contribution.

Here, we present measurements of the transverse and longitudinal sound speeds in polycrystalline cerium at higher temperatures than ambient. We find that the step-like increase in *G* at the VC decreases with temperature and disappears, and—for temperatures approaching the critical point—a negative Poisson’s ratio which makes elemental cerium auxetic under these conditions. The decrease in the bulk modulus *B*_S_ approaching the VC appears to be caused solely by the decrease in the elastic constant *C*_12_. The lattice contribution Δ*S*_vib_ to the total entropy change Δ*S* becomes less important with temperature while both vanish at the critical point just like the intrinsic hysteresis. Other mechanisms must then fill the widening gap in Δ*S*, such as increasing thermal disorder.

## Results

### Ultrasonic velocities

**Fig. 1:**
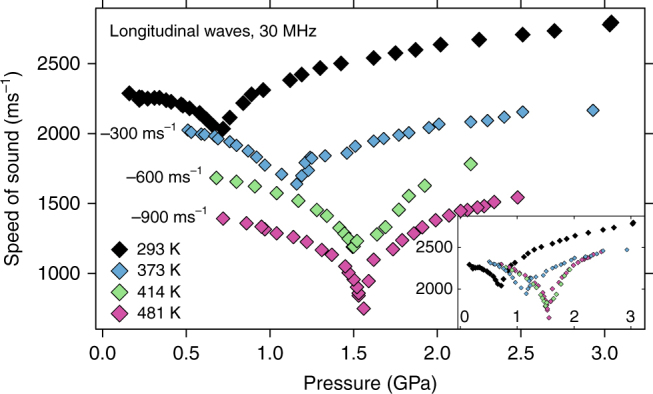
Ultrasonic longitudinal velocities. The values at 293 K, 373 K, 414 K, and 481 K are displayed with an offset by subtracting 300 ms^−1^ (373 K), 600 ms^−1^ (414 K) and 900 ms^−1^ (481 K) for better clarity. The inset shows the same data without offsets. Uncertainty in pressure (<0.05 GPa) and speed (<1%) is within symbol size

**Fig. 2:**
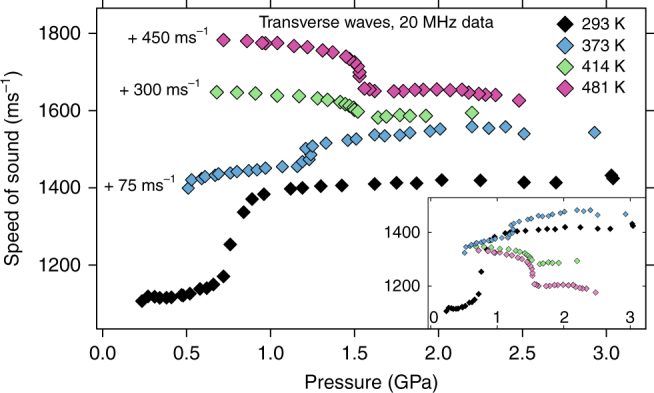
Ultrasonic transverse velocities. The values at 293 K, 373 K, 414 K, and 481 K are displayed with an offset by adding +75 ms^−1^ (373 K), +300 ms^−1^ (414 K), and +450 ms^−1^ (481 K) for better clarity. The inset shows the same data without offsets. Uncertainty in pressure (<0.05 GPa) and speed (<1%) is within symbol size

Cerium shows some unexpected behavior of the longitudinal and transverse velocities *c*_L_ and *c*_T_ with pressure. The values for the ultrasonic velocities are displayed in Figs. 1 and 2 for temperatures of 293, 373, 414, and 481 K, with 481 K very close to or at the critical temperature^8–11^.

Looking at the 373, 414, and 481 K isotherms in Fig. 1 for the longitudinal sound speeds one finds that they all start out at ~2300 ms^−1^ when the pressures are far from the volume collapse pressure *P*_VC_ which increases with temperature^18^. At higher temperature, c_L_ decreases even more approaching the transition pressure, at 481 K down to 1600 ms^−1^. After the VC in the α-phase, all wave speeds increase again about as quickly as they decreased.

Figure 2 shows the completely different behavior of the transverse waves. They either climb (373 K) or decrease slightly (414 and 481 K) with pressure far away from the transition in each phase but show a pronounced increase (373 K) or decrease (414 and 481 K) at the transition pressure. Also, at 373 K, the transverse wave speed in the γ-phase has already exceeded the transverse wave speeds observed at RT previously by others^5, 25, 26, 28^ but does not change much further with temperature up to 481 K.

A detailed comparison to RT data of other authors has been published previously^27^. While all the RT data for the longitudinal sound speed are in very good agreement in the γ-phase, the values for the transverse wave speeds are somewhat different with the ones in this study being ~200 ms^−1^ lower. Beyond the VC the transverse values are again in agreement^25–27^, which indicates that the present sample environment^28^ provides trustworthy data. The possible reason for the transverse sound speed difference in the γ-phase has been discussed at length in ref. ^27^ before and is most likely caused by different sample treatment. The samples used in the present study have not been subjected to long periods of heating^5^ or even melting and subsequent quenching^26^. In fact, our values for the transverse speed lead to a much closer agreement with previously published Debye temperature data obtained by measurements of the complete phonon dispersion relation in cerium under pressure by either inelastic neutron or X-ray scattering on single crystals^27, 32, 33^.

### Adiabatic bulk modulus *B*_S_ and shear modulus *G*

**Fig. 3:**
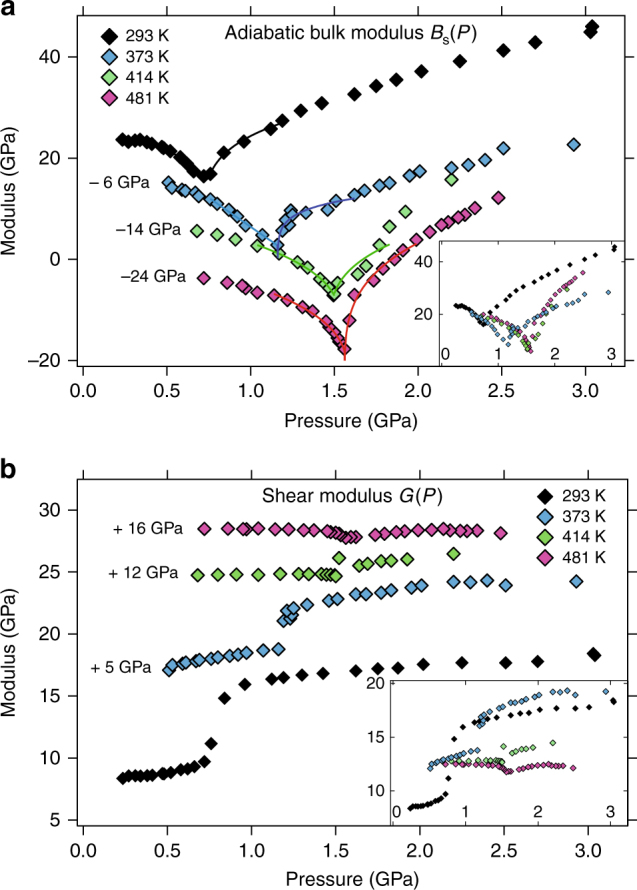
Pressure dependence of the adiabatic bulk and shear moduli. **a** Values for the adiabatic bulk modulus *B*_S_ = *ρ*(*c*_L_^2^−4/3*c*_T_^2^) are shown for different isotherms. Width and depth of the transition for *B*_S_ become narrower and deeper with the temperature approaching the critical temperature *T*_C_. A similar behavior has been observed for the isothermal bulk modulus *B*_T_ above *T*_C_ where the width of the transition becomes wider and the minimum shallower with further distance from *T*_C_. At *T*_C_ itself *B*_T_ vanishes at the critical pressure *P*_C_^10, 11^. The solid lines are the fits of a power law *B*_S_(*P*) ~ |*P*−*P*_VC_|^*α*^ to the data. **b** Values for the shear modulus *G* = *ρc*_T_^2^ at different temperatures. Uncertainty in pressure (<0.05 GPa) and modulus (<3%) is within symbol size

With the previously measured densities for the chosen temperatures^10^ we are able to obtain values for the shear modulus *G* = *ρc*_T_^2^ and adiabatic bulk modulus *B*_S_ = *ρ*(*c*_L_^2^ − 4/3*c*_T_^2^) under pressure (Fig. 3a). The minimum of the bulk modulus *B*_S_ at the VC at RT continues at higher temperatures with a deeper and steeper minimum. As for the shear modulus *G*, cerium shows an initial increase in the γ-phase with temperature—contrary to the usually observed decrease in metals. Under pressure, *G* rises slightly in the γ-phase and then increases step-like across the VC from the γ-phase to the α-phase but this step vanishes with temperatures closer to *T*_C_. At the critical point Ce becomes a solid without compressive strength (*B*_T_ = 0) but finite shear strength.

The adiabatic bulk modulus decreases in the γ-phase for all temperatures until it reaches its minimum at the VC transition and starts rising again continuously in the α-phase. With higher temperature, the minimum moves to a higher pressure and becomes deeper. Since *B*_T_ = 0 at the critical point^10, 11^ and *B*_S_ = *B*_T_ *C*_P_/*C*_V_ it follows that *B*_S_ should fall to zero at the critical point just as well since *C*_P_ and *C*_V_ are nonzero^34, 35^. Our lowest observed value is *B*_S_ ~ 6 GPa for *p* = 1.55 GPa at 481 K which could mean that our temperature was just barely different from the critical temperature. It is possible to fit the pressure dependence of *B*_S_ near the transition to a power law *B*_S_ (*P*) ~ |*P*−*P*_VC_|^*α*^ ^4, 26^. The solid lines in Fig. 3a show the resulting fits to the data. The values for *P*_VC_ and *α* are listed in Table 1 for a pressure range of about 0.4 GPa below (−) and above (+) the transition. However, except for the critical temperature, the transition already takes place at *P* < *P*_VC_. This was observed previously by X-ray diffraction for *B*_T_^4^ and ultrasonic measurements for *B*_S_^26^ at room temperature which found *α* ^−^ = 0.46, *P*_VC_^−^ = 0.83 GPa^4^ and *α* ^−^ = 0.42, *P*_VC_^−^ = 0.92 GPa^26^, respectively. At the critical temperature (481 K) we find *α* ^−^ = 0.26 and *α* ^+^  = 0.28, basically equal within the experimental uncertainty. An exponent of 0.5 would indicate Gaussian fluctuations^4^ associated with electron–phonon coupling^26^, an exponent of zero is expected in the mean-field theory. Our value of *α* = 0.27 ± 0.01 is the only one taken approximately on the critical isotherm which allows a straight forward interpretation. All other values were obtained at lower temperatures for which the descent of the bulk modulus towards zero is interrupted by the transition and thus the measured exponents are not truly “critical”^4^. Additionally, the further the temperature is from *T*_C_ the larger is the gap in the transition pressures *P*_VC_^−^ and *P*_VC_^+^ which we list in Table 1 as Δ*P*. This value can serve as a measure of hysteresis that disappears approaching the critical temperature. Table 1 also provides a comparison with previously published results and includes values for fits to the data of refs. ^5, 25^ that were obtained within the current effort. With the exception of the 481 K isotherm, all exponents measured on the lower pressure side are larger than the ones from the higher-pressure side which one would expect similarly for the behavior of a van der Waals liquid/gas near the critical point.

**Table 1:** Pressure *P*_VC_ (GPa) and exponent *α* obtained by fitting *B*_S_(*P*) ~ |*P*−*P*_VC_|^*α*^ to the data

T (K)	*P*_VC_^−^, *P*_VC_^+^ (GPa)	*α* ^−^, *α* ^+^	Method
289	0.89(3), 0.49(4) Δ*P* = 0.4	0.27(2), 0.11(3)	(a) Ultrasound^a^
293	0.79(5), 0.61(16) Δ*P* = 0.18	0.35(7), 0.3(4)	(b) Ultrasound^a^
RT	0.83, −	0.46, −	(c) X-ray diffraction
RT	0.92, −	0.42, −	(d) Ultrasound
293	0.89(11), 0.71(3) Δ*P* = 0.18	0.34(13), 0.20(5)	Present work, ultrasound
373	1.25(11), 1.16(1) Δ*P* = 0.09	0.50(17), 0.14(2)	Present work, ultrasound
414	1.53(1), 1.47(4) Δ*P* = 0.06	0.34(2), 0.32(8)	Present work, ultrasound
481	1.55(1), 1.56(1) Δ*P* = 0.01	0.26(2), 0.28(3)	Present work, ultrasound

The superscripts − and + refer to the results for the low (−) and high pressure (+) fitting range

(a) ref. ^5^ (b) ref. ^25^ (c) ref. ^4^ (d) ref. ^26^

^a^Fit to previously published data performed within present work

Based on their RT data Jeong et al.^4^ stated that the drop in *B*_T_ towards the VC follows directly from the decrease of the *C*_11_ elastic constant since *B* = 1/3[*C*_11_ + 2*C*_12_] = 1/3[3*C*_11_−4C*] for a cubic lattice and the shear modulus C* = (*C*_11_ − *C*_12_)/2 would be insensitive to pressure^5^. This assumption, however, is not warranted since C* is just one of the principal shear moduli, the other one being *C*_44_. The behavior within each phase of the relatively pressure-insensitive shear modulus *G* (Fig. 3b) for polycrystalline cerium in the Voigt or Reuss limit is dominated by *C*_44_ since *C*_44_ is unusually large. *C*_44_ (19.4 GPa at ambient pressure) amounts to 67–80% of the value of *C*_11_^33, 36^, a property not found to that extent in any other element except δ-Pu^33, 37^. Indeed, at *p* = 0.8 GPa, *C*_44_ > *C*_11_^32^. Moreover, Krisch et al. state that the phonon dispersion relations in general do not evolve strongly under compression in the γ-phase but change significantly at the location of the γ–α transition^32^. Close inspection of the phonon dispersion relations of cerium under pressure published by them^32^ shows that *C*_11_ does not decrease under compression. This leaves only *C*_12_ as the elastic constant responsible for the pressure dependence of *B* = 1/3 (*C*_11_ + 2*C*_12_). *C*_12_ is already much smaller than *C*_44_ at ambient conditions (Stassis et al.^33^ report *C*_12_ = 10.2 GPa for γ-Ce at RT) and its decrease therefore must be the only source for the pressure dependence of *B*. *C*_12_ does not participate in waves propagating in the [100] direction, but affects the wave speed in the [110] and [111] direction, in expressions that also contain *C*_11_ and/or *C*_44_^38^, consistent with the highly directional character of the *f*-orbital bonding. At RT, *B* does not drop all the way to zero since the acoustic phonon frequencies do not soften.

Additionally, at RT, *C*_12_ itself does not vanish under pressure since in that case *B*_S_ would drop to ~7 GPa which is clearly not seen in our or others’ experiments^5, 26, 28^. In principle, however, a negative *C*_12_ would not be prohibited as has been observed for intermediate valent compounds of 4 *f* metals like TmSe or Sm_*x*_La_1−*x*_S^39, 40^. While small, the effect of *C*_12_ on bulk and shear modulus is not negligible. Since the bulk modulus is the same for the Voigt and Reuss limits, *B* = *B*_V_ = *B*_R_ = 1/3 (*C*_11_ + 2*C*_12_) = 0 at the critical point (~1.5 GPa and 480 K), it follows that *C*_12_ = −1/2 *C*_11_. At the critical point *C*_12_ is indeed negative and exactly half the size of *C*_11_.

*C*_12_ enters both shear modulus limits: *G*_V_ = 1/5(*C*_11_−*C*_12_ + 3*C*_44_) in the Voigt limit assuming constant strain and in the Reuss limit assuming constant stress *G*_R_ = 5(*C*_11_−*C*_12_) *C*_44_/[4*C*_44_ + 3(*C*_11_−*C*_12_)]. This can be simplified even more since *C*_44_ becomes larger than *C*_11_ at a pressure of 0.8 GPa^32^ at RT and therefore both must be approximately equal at the pressure of the volume collapse. If we make this assumption that *C*_44_ ≈ *C*_11_ = −2 *C*_12_ = *C* at the critical point, we can simplify the expressions for *G*_V_ and *G*_R_ to G_V_ = 0.9 *C* and *G*_R_ = 15/17 *C* ≈ 0.88 *C*. The real *G* should fall between the very narrow gap between *G*_V_ and *G*_R_. *G* is finite and non-vanishing. Under the same assumptions the anisotropy ratio *A* = *C*_44_/½(*C*_11_−*C*_12_), which is 2.8 at ambient conditions, decreases to about *A* ≈ 4/3 at the critical point.

The pressure insensitivity of the acoustic phonons in Ce was already anticipated by Entel and Grewe^41^ in the context of their enhanced periodic Anderson model for cerium which was confirmed experimentally in X-ray scattering experiments^32^. Instead Entel et al. expected a softening of the longitudinal optical phonons when approaching the phase boundary^41^ which was not detected.

Figure 3b shows the remarkable behavior of the shear modulus *G* = *ρc*_T_^2^. Increasing the temperature to 373 K also increases *G* in both phases compared to 293 K but the step-like increase at the transition now amounts to only about half of the step-like transition at 293 K. Within both phases *G* rises slightly by about 2 GPa/GPa. At 414 K the step at the transition becomes even smaller (about 1 GPa) and for 481 K the step has essentially vanished with the exception of a small dip at a pressure of 1.56 GPa. At 414 K there is almost no change under pressure within each phase and the data at 481 K show a very slight decrease in *G* with pressure. At ~0.8 GPa pressure the shear modulus remains basically constant for all temperatures at ~12.5 GPa. In contrast, the behavior of a normal metal would have *G* slightly increasing with pressure and decreasing with temperature.

We established above—under the assumption that *C*_44_ ≈ *C*_11_ at the critical point—G_V_ = 0.9 *C* and *G*_R_ ≈ 0.88 *C*. Since *G* = 12 GPa under these conditions it follows that *C*_44_ ≈ *C*_11_ ≈ 13.5 GPa and *C*_12_ ≈ −6.8 GPa.

### The Poisson’s ratio *ν*

**Fig. 4:**
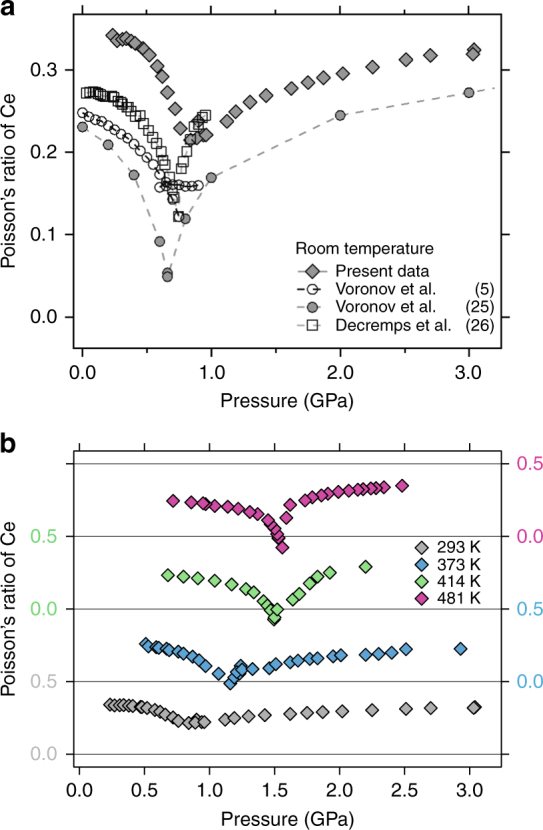
The Poisson’s ratio *ν* = (3*B*−2 *G*)/(6*B* + 2 *G*) versus pressure. **a** Comparison of ambient temperature values. Values from refs. ^25, 26^ are calculated from their published results for *B* and *G*. **b** The Poisson’s ratio versus pressure for different temperatures. The horizontal lines represent values of 0 and 0.5. Towards *T*_C_ the width of the transition decreases and the minimum becomes deeper and negative. Uncertainty in pressure is <0.05 GPa and the symbols for the present data are larger than the error bars

The ratio of *B*/*G* determines the material response of transverse strain δ*ε*_T_ to longitudinal strain *δ* *ε*_L_ via the Poisson’s ratio *ν* = −*δ* *ε*_T_/*δ* *ε*_L_ = (3*B*−2*G*)/(6*B* + 2 *G*). The Poisson’s ratio becomes 0.5 for liquids (*G* = 0) which serves as a diagnostic for melting in shock wave experiments of cerium, iron and others^42–44^. A comparison of Poisson’s ratios at RT with previously published results is shown in Fig. 4a. While the absolute values from different authors show a substantial spread in the γ-phase they all exhibit qualitatively the same features: a slight decrease with pressure is followed by a significant drop towards the VC. In the α-phase the Poisson’s ratios rise again. In the case of Voronov’s data from 1979^25^ *ν* drops to 0.05 at the VC.

Interestingly, *ν* can become negative for the right ratios of *B* and *G*, a feature that has been sought to exploit in engineering applications of other such materials^44, 45^. This turns out to be the case for cerium at the VC at higher temperatures closer to the critical point (Fig. 4b). For our temperatures of 373, 414, and 481 K (Fig. 4b), all the data show a drop of ν below 0 near the VC. This drop becomes deeper and steeper with increasing temperature. Since *B*_S_ should be zero at the critical point the Poisson’s ratio should drop down to −1 for (*P*_C_, *T*_C_). Our lowest observed value is ~−0.1 at *p* = 1.56 GPa. In other words, close to the VC, cerium metal becomes auxetic approaching the critical temperature, meaning an axial compression would result in a lateral contraction (or an axial elongation results in a lateral widening).

Cerium is unique in this regard, since the reason lies with the behavior of the 4 *f* electron configuration. Typically, auxetic materials have open structures like foams^46^ or α-cristobalite, a phase of SiO_2_^47^. Once considered an exotic and rare characteristic of materials it has been found that at least in certain specific crystallographic directions such as the [110], many cubic metals exhibit a negative Poisson’s ratio^45^. For cerium however, at elevated temperatures near the VC, there are no restrictions with regard to crystallographic directions and no open structures are required for one of the participating phases.

Traversing the transition from the α-phase to the γ-phase by temperature instead of pressure should show a very similar behavior of *ν*—a decrease towards the VC, possibly to negative values, followed by an increase back to similar values as they were before the VC. This behavior due to the *f*-electron configuration in cerium is therefore quite different from that of the 5 *f* metal Pu. The temperature dependence of the Poisson’s ratio in Pu shows a large discontinuous step-like increase (α–β phase transition) and then a decrease (β–γ phase transition)—over a temperature range of just 100 K as if the different phases were completely different metals^48^. Theoretically this has been attributed to an increase in *f*-electron correlation^49^. In cerium at ambient temperature the f-electron occupancy experiences a ~20–30% drop at the VC^50, 51^. The aforementioned cubic intermediate valence compounds Sm_*x*_La_1−*x*_S and TmSe also exhibit a negative Poisson’s ratio which is due to a negative *C*_12_ elastic constant^39, 40^.

### Debye temperature and lattice contribution

The knowledge of *c*_L_ and *c*_T_ also allows us to estimate the lattice contribution to the total entropy change across the VC for different temperatures. As it turns out the lattice contribution vanishes faster than the total entropy itself suggesting that other mechanisms need to come into play to stabilize the γ-phase against the α-phase. Increasing thermal disorder could be a candidate.

**Fig. 5:**
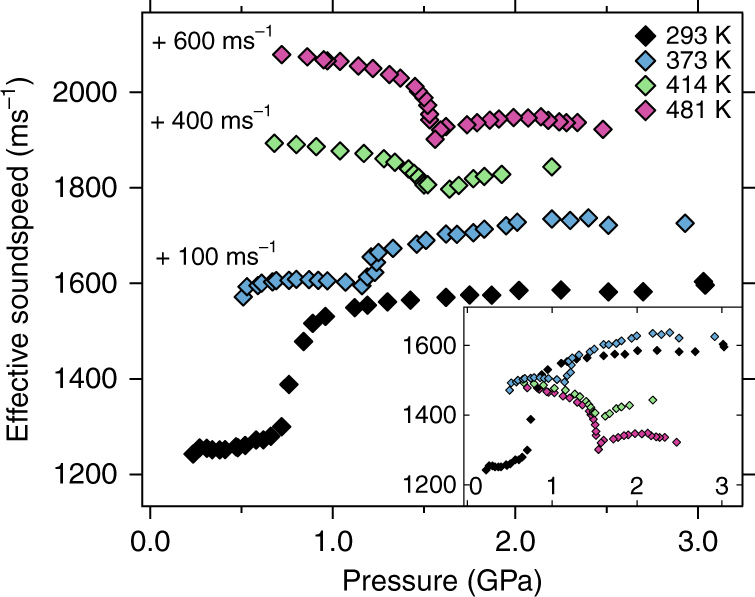
Effective sound speed in cerium. The effective sound speed is a measure of the stiffness of the crystal^15^ and is shown versus pressure (uncertainty < 0.05 GPa) at 293 K, 373 K, 414 K, and 481 K. Curves individually offset for clarity. Symbols are larger than the experimental uncertainties

Figure 5 displays the effective sound speed which is needed for the calculation of the Debye temperature *θ*_D_(*p*). Knowledge of the change of the Debye temperature across the VC allows a determination of the lattice contribution to the total entropy change. *C*_eff_ is obtained from *c*_T_ and *c*_L_ according to (e.g., ref. ^52^):

1 $$C_{{\mathrm{eff}}} = \left( {\frac{1}{3}\left( {\frac{2}{{c_{\mathrm{T}}^3}} + \frac{1}{{c_{\mathrm{L}}^3}}} \right)} \right)^{\!\! - \frac{1}{3}}$$

*C*_eff_ offers an assessment of the stiffness of the crystal^15^. At 293 K and 373 K it is lower in the γ-phase than in the α-phase but this behavior reverses for 414 and 481 K. At 293 K the increase amounts to more than 25% but reduces to <5% at 373 K. At 414 K *C*_eff_ drops from the γ-phase to the α-phase by ~4% and by almost 10% at 481 K. The effective sound speed enters into the determination of the Debye temperature via:

2 $$\theta _{\mathrm{D}} = \frac{h}{{2k_{\mathrm{B}}}}  \cdot C_{{\mathrm{eff}}} \cdot \root {3} \of {{\frac{6}{{\pi \,V_{{\mathrm{at}}}}}}}\sim \frac{{C_{{\mathrm{eff}}}}}{a}$$

with *h* as Planck’s constant, *k*_B_ as the Boltzmann constant, *V*_at_ the atomic volume of a cerium atom and *a* as the lattice constant^52^. The change in the lattice constant going from the γ-phase to the α-phase is 5.1% at 293 K and less at higher temperatures.

**Fig. 6:**
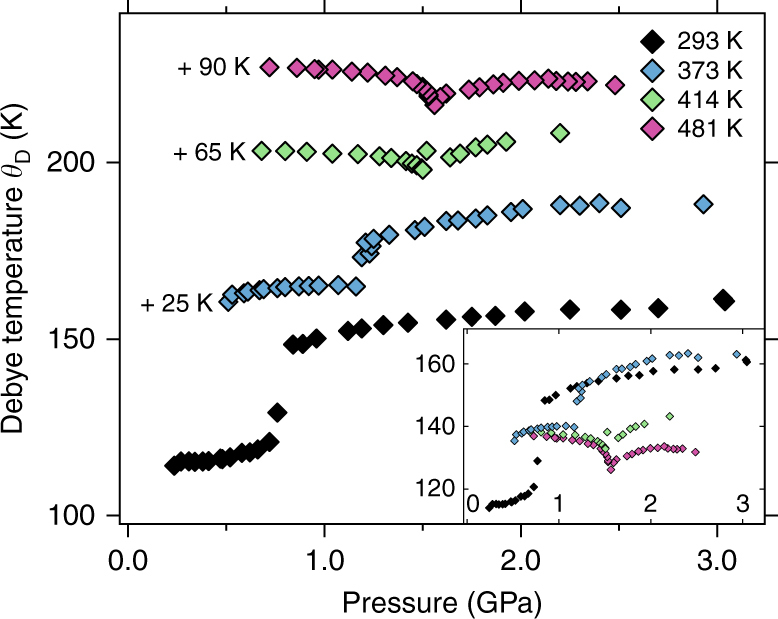
Debye temperatures of polycrystalline cerium. They are shown versus pressure (uncertainty < 0.05 GPa) at different temperatures. Symbols are larger than the experimental uncertainties

Figure 6 shows the Debye temperatures *θ*_D_ derived this way. At high temperatures these can be used to approximate the lattice contribution *∆S*_vib_ = 3 *k*_B_ ln(*θ*_α_/*θ*_γ_) to the total entropy change across the VC transition *∆S* = Δ*V*d*P*/d*T* ^4^. We used d*P/*d*T* = 5.14×10^6^ Jm^−3^K^−1^ ^27^. Table 2 lists all the involved quantities.

**Table 2:** Dependence of volume collapse parameters on temperature

*T* (K)	Δ*V* (Å^3^)	*∆S* in *k*_B_	*θ*_γ_ (K)	*θ*_α_ (K)	*∆S*_vib_ in *k*_B_	Ratio *∆S*_vib_/*∆S*
293	4.74	1.765	119	149	0.67	0.38
373	4.04	1.505	140	153	0.27	0.18
414	3.64	1.356	133	138	0.11	0.08
481	0	0	No discernible step		0	–

The last column of Table 2 shows that the contribution *∆S*_vib_ to the total entropy change becomes less important for higher temperatures. While it still supplies almost 40% at RT, that share drops to <20% at 373 K and to <10% at 414 K. Since there is general consensus that the γ–α transition is entropy driven^29–31, 53, 54^, another mechanism has to fill the growing gap in entropy and acquire more weight with temperature. One natural candidate is the increasing amount of thermal disorder. Indeed, Jarlborg^55^ has found via DFT calculations on large supercells that this disorder produces fluctuations in the magnitude of the magnetic moments leading to an increase in the average moment for the low pressure γ-phase. The resulting entropic contribution additionally stabilizes the γ-phase at high temperatures with respect to the α-phase^55^.

## Discussion

The VC phase transition in Ce starts with the nucleation and growth of many independent crystallites in the α-phase uniformly dispersed in the γ-phase based on the observation that a small diameter X-ray beam of 20 µm always detects both phases once the transformation starts^56^. The transformed regions remain in the same crystallographic orientation as the non-transformed material. This allows, for example, for a single crystal in the γ-phase to become a single crystal in the α-phase while dislocations and deformation bands are also created^56^. The ultrasonic waves in the current experiment probe regions that are even larger and are not sensitive to details on the atomic but on the macroscopic ( = averaged) scale. Indeed, due to the iso-structural nature of the collapse, the step-like change in the transverse velocity and shear modulus can be obtained by simply averaging the individual properties of the two distinct phases in the mixed-phase region according to their concentration.

Many of the current results are consistent with a non-linear elastic model of a discontinuous volume change advanced by Bustingorry et al^57^. Under the assumption of a coherent transformation (i.e., without taking structural defects into account) between two isotropic phases with the same bulk and shear modulus it is expected that the bulk modulus softens according to *B* ~ |*P*−*P*_Vc_|^*α*^ with *α* = ½ even at temperatures far away from the critical point. While our measured exponents are different from ½ (Table 1), a softening of the bulk modulus according to a power law is clearly observed. This also implies that the Poisson’s ratio becomes negative^57^, again experimentally observed by us.

The difference between *P*_VC_^−^ and *P*_VC_^+^ (Table 1) serves as a measure of the width of the transition^57^. Our experiment shows that this width shrinks with increasing temperature and disappears when the temperature approaches the critical value. This intrinsic hysteretic behavior is also reproduced in the model by Bustingorry et al.^57^ Not only does the bulk modulus vanish according to *B* ~ |*P*−*P*_VC+_|^*α*^ but it also jumps discontinuously to a finite value for *p* > *P*_VC_−. Previous experimental confirmation of this feature was seen in the data of Jeong et al.^4^ at room temperature. Additionally, we can now confirm that this intrinsic hysteresis disappears indeed when the system approaches the critical temperature.

While the model is based on specific assumptions that are conflicting with the properties of real materials (anisotropy and structural defects are not incorporated) the authors feel nevertheless that the features are robust enough to hold up under more general assumptions.^57^ A simple extension of the model would be to work with different values of bulk modulus and shear modulus on both sides of the transition.

## Methods

A detailed design of the sample assembly can be found in ref. ^27^. In summary, the 1.5 mm diameter and about 0.7 mm high polished Ce disks—polishing technique described in ref. ^58^—were made from commercially acquired foil (Alfa Aesar, at least 99.9% purity) of 1 mm starting thickness. The level of impurities compares well with other previous ultrasonic experiments that used cerium disks with purity levels ranging from 98.5%^5^, 99.53 to 99.93^25^ to 99.99%^26^. Impurities in our sample (Al, Fe, Mg, Ni, Si, Ca, La, Nd, Pr, and Y) are <0.01% each. For the ultrasonic measurements, the cylindrical sample was surrounded on the sides by crushable boron nitride (BN) and a MgO tube. This assembly was sitting inside another BN cylinder and mounted inside a graphite heater^59, 60^. The heater in turn was supported by another MgO ring pushed into amorphous boron epoxy. The outer region of the sample assembly was formed by a ring of lexan. The temperature in the sample region was determined via a previous calibration by thermocouples against power consumption^59, 60^ with an uncertainty of ~5 K for temperatures other than RT.

The ultrasonic pulses were transmitted to the top of the cerium disk through a cylinder of highly polished Al_2_O_3_. One part of the pulses was reflected back at this interface. Another disk of Al_2_O_3_ contacted the cerium disk on the bottom to reflect the sound waves from the bottom interface of the sample. The time difference between the two reflected pulses gave the travel time corresponding to twice the sample length. The pulse-overlap method—for details see ref. ^60^—was used to measure the travel time of the pulses through the sample to within nanoseconds resulting in ultrasonic velocity values with <1% error. The frequency of the ultrasound pulses was varied between 15 and 30 MHz since better coupling to the transverse waves was achieved at the lower frequency range, and for the longitudinal ones at 30 MHz.

Thickness measurement of the sample to within microns in situ was provided by X-ray radiography^60, 61^. The X-ray beam (dimensions of ~1 mm × 1 mm) produced a radiographic image on a thin scintillator crystal whose visible luminescence was focused onto a CCD camera by a microscope objective^27^. The length per pixel was precisely calibrated.

Energy dispersive X-ray diffraction (taken close to the outer edge of the cerium disk since the center thickness of 1.5 mm was opaque to the X-rays) provided the pressure using the previously established EOS^10^. Most importantly, the frequent collection of X-ray diffraction data allowed the assurance that the—with temperature—increasingly reactive sample remained elemental cerium. Indeed, we did not observe any signs of reactivity during our measurements, a problem which was previously encountered in experiments much closer to the melt^13^.

The quasi-hydrostatic sample environment in our set-up is adequate for an accurate measurement of the transverse and longitudinal sound velocities under pressure. Experimental validation of this fact was provided by an earlier experiment on SiO_2_ glass that found no difference between the values for the transverse and longitudinal sound velocities obtained using the present set-up and others^60^. Additionally, the volume collapse transitions take place in an identical fashion to previous reports for polycrystalline cerium compressed in a diamond anvil cell. Those used either NaCl or helium as pressure transmitting medium^4, 10, 11^, also with no apparent difference in sample behavior. Our data begin at about 0.25 GPa or higher since the coupling between ultrasonic waves and sample was not efficient enough at lower pressures.

All the experiments took place at the 16 BMB beam-line (HPCAT) at the Advanced Photon Source (APS) at Argonne National Lab. An important difference between our present technique and other previous ultrasonic measurements is that the sample characteristics were directly measured in situ throughout the experiment: There was no need for further assumptions and iterative schemes that would have required exact knowledge of additional thermodynamic quantities.

### Data availability

The data that support the findings of this study are available from the corresponding author upon request.
